# Supplementary material for: Ecdysteroid Biosynthesis Halloween Gene Spook Plays an Important Role in the Oviposition Process of Spider Mite, Tetranychus urticae
Source: Int J Mol Sci. 2023 Sep 30;24(19):14797. doi: 10.3390/ijms241914797 (PMC10573261; doi:10.3390/ijms241914797)
Supplement: Supplementary file 1 [file ijms-24-14797-s001.zip › ijms-2595460-supplementary.pdf]

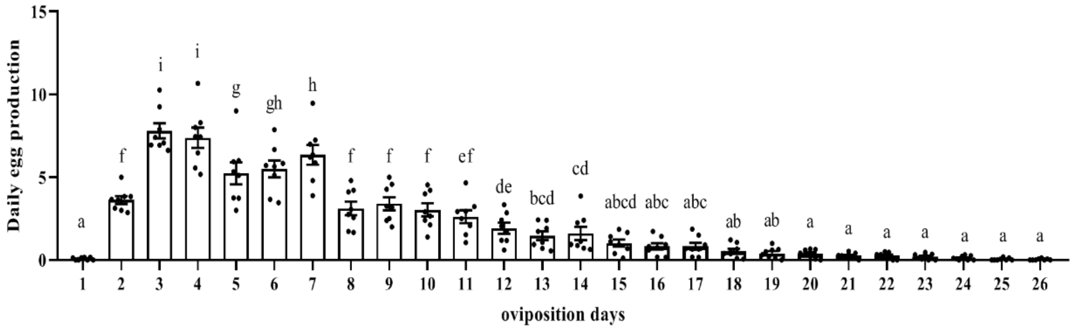

Figure S1: The average daily egg production of female adult mites of *T. urticae* during their whole oviposition period. The results are presented as mean (±SE) based on eight biological replicates per day. Lowercase letters above each column indicate significant differences amongst the ten days using one-way analysis of variance (ANOVA) followed by Tukey's honestly significant difference test ($p < 0.05$).

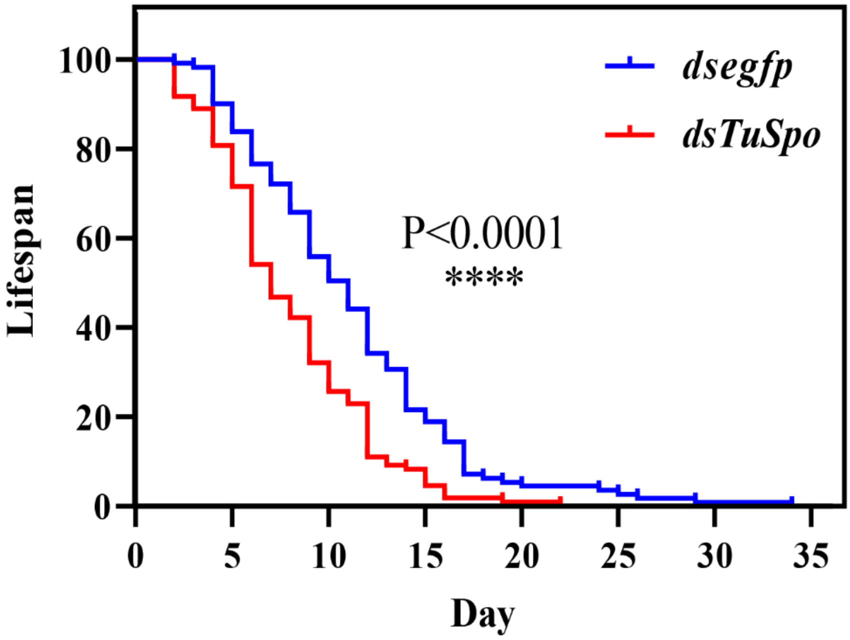

Figure S2: The Effects of ds*TuSpo* injection in female mites of *T. urticae* on the lifespan of their offspring. Significant difference between the two groups were indicated with '****', p<0.0001, after a Student's t-test.

Table S1. The development of offspring of the female mites injected with dsRNA of ds*egfp* and ds*TuSpo*

Mite State	Experience Days (mean ± SE) (d)		P.value	Significance
	ds*egfp*	ds*TuSpo*		
Egg	4.53 ± 0.07	4.67 ± 0.10	0.3195	ns
Larva	1.68 ± 0.04	1.75 ± 0.11	0.5395	ns
Protonymph	1.43 ± 0.03	1.67 ± 0.10	0.0518	ns
Deutonymph	1.71 ± 0.03	1.83 ± 0.10	0.2807	ns
